# Febrile infection-related epilepsy syndrome with claustrum sign following a human rhinovirus-positive respiratory illness in an adult: a case report

**DOI:** 10.3389/fimmu.2026.1935305

**Published:** 2026-09-17

**Authors:** Tiejun Yang, Longnan Wang, Chunbo Ji, Siqing Ma, Danyang Dong, Ping Yang

**Affiliations:** 1 The First Clinical Medical College of Ningxia Medical University, Yinchuan, Ningxia, China; 2 Department of Neurology, General Hospital of Ningxia Medical University, Yinchuan, Ningxia, China

**Keywords:** electroencephalography, febrile infection-related epilepsy syndrome, magnetic resonance imaging, rhinovirus, tocilizumab

## Abstract

**Background:**

Human rhinovirus (HRV) is the predominant viral pathogen causing community-acquired upper respiratory tract infections, typically manifesting as mild coryzal symptoms. Severe neurological complications, including febrile infection-related epilepsy syndrome (FIRES), are exceptionally rare.

**Case presentation:**

We report a case of febrile infection-related epilepsy syndrome (FIRES) with bilateral claustral involvement occurring in temporal association with an HRV-positive febrile respiratory illness, identified at the General Hospital of Ningxia Medical University in April 2025. The patient presented with acute refractory status epilepticus following a prodromal febrile illness. Neuroimaging revealed bilateral claustral hyperintensities and symmetrical thalamic lesions, while continuous electroencephalographic monitoring demonstrated burst-suppression patterns with frontotemporal discharges. HRV was detected in a respiratory tract specimen by nucleic acid testing but was not detected in the cerebrospinal fluid; the relationship between HRV detection and the neurological syndrome is therefore interpreted as a temporal association rather than a proven causal one, since a single respiratory detection may represent asymptomatic carriage rather than active infection.

**Conclusions:**

This case underscores the importance of considering FIRES in patients with acute encephalopathy following a respiratory infection, even when the implicated pathogen—human rhinovirus in this case—is detected only in the respiratory tract, where it may represent either active infection or asymptomatic carriage. Early recognition through comprehensive neuroimaging and electroencephalographic monitoring, combined with multimodal immunomodulatory and antiseizure therapy, was followed by clinical and electrographic improvement and a favorable functional outcome in this patient; however, because corticosteroids, intravenous immunoglobulin, tocilizumab, a ketogenic diet, multiple antiseizure medications, and intravenous anesthetics were administered concurrently or sequentially, the contribution of any specific treatment cannot be determined, and no conclusions regarding treatment efficacy or the clinical utility of cytokine monitoring can be drawn from this single case. The hypothesized cytokine-mediated mechanisms underlying this clinico-radiological entity are discussed in a narrative review of the literature.

## Introduction

Human rhinovirus (HRV; family Picornaviridae, genus Enterovirus) is the predominant cause of community-acquired upper respiratory tract infections worldwide. It is typically self-limited and exceptionally rarely causes neurological complications; reports of HRV-associated central nervous system infection remain exceedingly scarce. To our knowledge, febrile infection-related epilepsy syndrome (FIRES) with claustral involvement temporally associated with an HRV-positive febrile respiratory illness has not been reported. We describe what we believe to be the first such adult case—bilateral claustral lesions following an HRV-positive febrile respiratory illness in a patient admitted to the General Hospital of Ningxia Medical University on April 6, 2025—and review the pertinent literature on pathophysiology, EEG features, ancillary investigations, and treatment.

## Case presentation

A 25-year-old female was admitted to our Neuro-intensive Care Unit in April 2025 with a chief complaint of “fever for 8 days, worsening with limb convulsions and altered consciousness for 5 days.” Taking the first day of fever (March 29, 2025) as illness day 1, seizures began on illness day 4 (3 days after fever onset), and admission on April 6, 2025, corresponded to illness day 9 (hereafter hospital day 1). The fever peaked at 40.3 °C, with generalized fatigue and poor appetite. The seizures comprised sudden loss of consciousness, leftward conjugate gaze deviation, facial cyanosis, tongue biting, and tonic posturing of the left extremities with perioral twitching; each episode lasted about one minute, without recovery of awareness between seizures, and she experienced more than 10 episodes. Her past medical and family history was unremarkable. At a local tertiary hospital, lumbar puncture showed a leukocyte count of 17/mm³; despite endotracheal intubation, mechanical ventilation, antipyretics, empirical antimicrobials, and antiseizure medications, her neurological status did not improve, and she was transferred to our institution.

Upon admission, the patient was deeply comatose (Glasgow Coma Scale 6T: E1, VT, M4) under continuous intravenous midazolam and remifentanil sedation. Both pupils were approximately 4 mm with sluggish light reflexes; there was no motor response to painful stimulation, muscle tone was diffusely decreased, deep tendon reflexes were absent bilaterally, plantar responses were neutral, and meningeal signs were absent. Complete blood count, thyroid function, purified protein derivative testing, TORCH panel, cranial computed tomography (CT), and inherited metabolic screening were unremarkable; chest CT showed bilateral pulmonary inflammatory infiltrates. Toxic and other etiologies were additionally excluded in a systematic manner (**Supplementary Table S3**): no history of alcohol misuse, recreational substance use, or toxic exposure was elicited, and, together with the unremarkable investigations detailed above and the comprehensive work-up summarized in **Supplementary Table S3**, no toxic, metabolic, or structural cause was identified. An epilepsy gene panel (target region capture with DNBSEQ-T7 sequencing, proband only) detected no clinically relevant single-nucleotide, copy-number, or mitochondrial variants. Lumbar puncture revealed an opening pressure of 230 mmH_2_O; cerebrospinal fluid (CSF) biochemistry was unremarkable, CSF cytology showed a leukocyte count of 12×10^6^/L, and CSF immunoglobulin G was 22.10 mg/g. Blood cultures were negative.

Respiratory tract and CSF specimens were tested for viral pathogens in parallel; the timing, assay methods, and results of all microbiological investigations are summarized in **Supplementary Table S1**. Both the nasopharyngeal swab (collected on hospital day 1, illness day 9) and a sputum specimen were tested with the same commercial multiplex real-time fluorescent reverse-transcription PCR assay (Respiratory Six-Virus Nucleic Acid Detection Kit; Shanghai BioGerm Medical Technology Co., Ltd., Shanghai, China), which simultaneously detects human rhinovirus and five other respiratory viruses—human parainfluenza virus types 1 and 3, human adenovirus, human metapneumovirus, and respiratory syncytial virus—in a two-tube format (results interpreted as positive when Ct ≤ 35 with a typical sigmoidal amplification curve, equivocal when Ct 36–38 and retested, and negative when Ct > 38 or undetected). The nasopharyngeal swab was positive for HRV and negative for the other five panel viruses; the sputum specimen, obtained in parallel on hospital day 1, was negative for all six panel viruses (including HRV). Of note, detection of HRV in a single upper respiratory tract specimen cannot distinguish between active infection and asymptomatic carriage, because respiratory shedding may persist for several weeks; this finding was therefore interpreted only as evidence of an HRV-positive febrile respiratory illness temporally associated with—rather than causally linked to—the neurological syndrome.

In contrast, CSF testing revealed no evidence of viral infection. A targeted next-generation sequencing (tNGS) assay was performed on the CSF (Common Pathogens Targeted Identification Panel, 400+ targets and drug-resistance genes; Wuhan Kindstar Zhenyuan Medical Laboratory, Wuhan, China): after nucleic acid extraction, pathogen-specific gene sequences were amplified and enriched by ultra-multiplex PCR and sequenced on a high-throughput sequencing platform, and the resulting reads were compared against microbial reference genome databases. The panel simultaneously interrogates 429 organisms—Gram-positive bacteria (114 species), Gram-negative bacteria (83 species), fungi (71 species), DNA viruses, RNA viruses (66 species), and parasites—together with antibiotic-resistance and virulence genes, with a lower limit of detection of 100 copies/mL; workflow quality controls include library-concentration checks, anti-contamination barcoding, internal controls, and negative controls. Its viral coverage includes the principal neurotropic agents—herpes simplex viruses 1 and 2, varicella-zoster virus, Epstein-Barr virus, cytomegalovirus, human herpesviruses 6–8, enteroviruses A–D, and echoviruses—as well as rhinoviruses A–C; rhinovirus was thus specifically interrogated, rather than relying on a generic pan-enterovirus PCR assay, whose primers do not reliably detect rhinoviruses. No pathogenic microorganisms, grey-zone signals, or background microorganisms were reported, and no resistance or virulence genes were identified. Separate singleplex PCR assays for individual neurotropic viruses were not performed; these agents were instead assessed within the panel’s viral coverage. Because this panel has not been formally validated specifically for CSF specimens and pathogens present below 100 copies/mL may escape detection, the negative results cannot exclude trace virus. Viral load and cycle threshold (Ct) values were not reported by this method, and HRV typing was not performed.

Autoantibody testing was performed on paired serum and CSF specimens collected on hospital day 2 and analyzed by cell-based assay (CBA) with fluorescence microscopy at an external reference laboratory (Beijing Hightrust Diagnostics Co., Ltd., Beijing, China; reported the following day). A 16-item autoimmune encephalitis antibody panel was tested—including anti-GABA-A receptor (α1 and β3 subunit) and anti-DPPX antibodies, specifically relevant because of their characteristic association with multifocal cortical/subcortical lesions and refractory status epilepticus, and including several antibodies associated with paraneoplastic neurological syndromes (anti-AMPAR1/2, anti-GABABR, anti-CASPR2, anti-IgLON5, anti-DPPX, anti-D2R, anti-GAD65, anti-GlyRα1, anti-mGluR1, anti-mGluR5)—together with a 3-item panel of demyelination-associated and astrocytopathy-related antibodies (anti-AQP4, anti-MOG, and anti-GFAP). All 19 antibodies were negative in both serum and CSF; the complete list, with methods and results, is provided in **Supplementary Table S2**. Screening serum tumor markers—carcinoembryonic antigen (CEA), alpha-fetoprotein (AFP), carbohydrate antigen 19-9 (CA19-9), CA72-4, CA125, human epididymis protein 4 (HE4), and CA15-3—were all negative, and chest CT showed no evidence of tumor, further arguing against a paraneoplastic etiology.

CSF cytokine analysis (sampled on the morning after admission, hospital day 2), performed by cytometric bead array on a BD FACSCanto II flow cytometer (BD Biosciences; nine cytokines), showed IL-1β (14.38 pg/mL; reference upper limit 12.4 pg/mL), IL-6 (121.93 pg/mL; upper limit 5.4 pg/mL), and IL-12p70 (4.16 pg/mL; upper limit 3.6 pg/mL) were elevated, whereas the other six cytokines were within their reference intervals or below the detection limit of 2.44 pg/mL. Serum IL-6 was 97.1 pg/mL (**Figure 1**).

**Figure 1:**
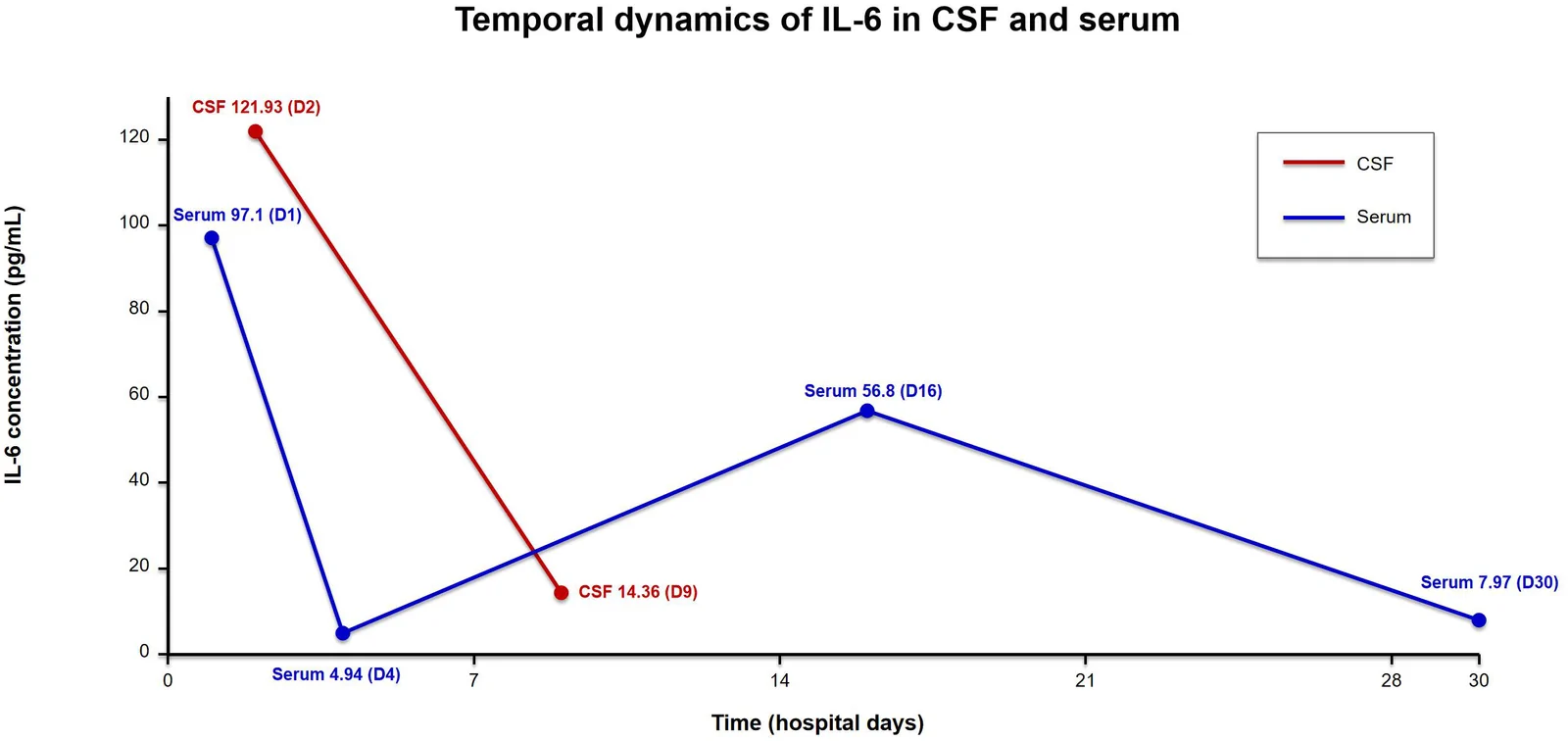
Temporal dynamics of IL-6 in cerebrospinal fluid (CSF) and serum. Red line, CSF; blue line, serum. CSF IL-6 was markedly elevated on day 2 (D2; 121.93 pg/mL) and declined to 14.36 pg/mL by D9. Serum IL-6 peaked at 97.1 pg/mL on D1, fell to 4.94 pg/mL by D4, rebounded to 56.8 pg/mL on D16, and normalized to 7.97 pg/mL by D30. The discordant kinetics suggest asynchronous intrathecal and systemic inflammation. CSF, cerebrospinal fluid; IL-6, interleukin-6.

Brain magnetic resonance imaging (MRI) performed on hospital day 2 (D2) revealed bilateral symmetric signal abnormalities in the thalami and claustra: isointense on T1-weighted imaging, mildly hyperintense on T2-weighted imaging and T2 fluid-attenuated inversion recovery (T2-FLAIR), and hyperintense on diffusion-weighted imaging (DWI) (**Figures 2A–D**). Continuous electroencephalographic (EEG) monitoring was initiated on admission: the first recording (hospital day 2) demonstrated a burst-suppression pattern. By hospital day 4, the background was predominantly delta activity with fast frequencies, with sharp-and-slow-wave and polyspike-and-slow-wave discharges over bilateral frontal and temporal regions, a burst-suppression tendency, and quasi-periodic discharges. Frequent electrographic seizures arose independently from bilateral frontal and temporal regions (right-predominant), as focal-onset or focal to bilateral tonic–clonic seizures lasting 1–2 minutes—findings consistent with electroclinical status epilepticus (**Figure 3A**). At the time of these recordings the patient was receiving multiple anesthetic and antiseizure agents (detailed below; **Figure 4**), which can themselves induce or deepen burst-suppression; the EEG findings should therefore be interpreted as observed under concurrent anesthetic treatment rather than as purely disease-intrinsic patterns.

**Figure 2:**
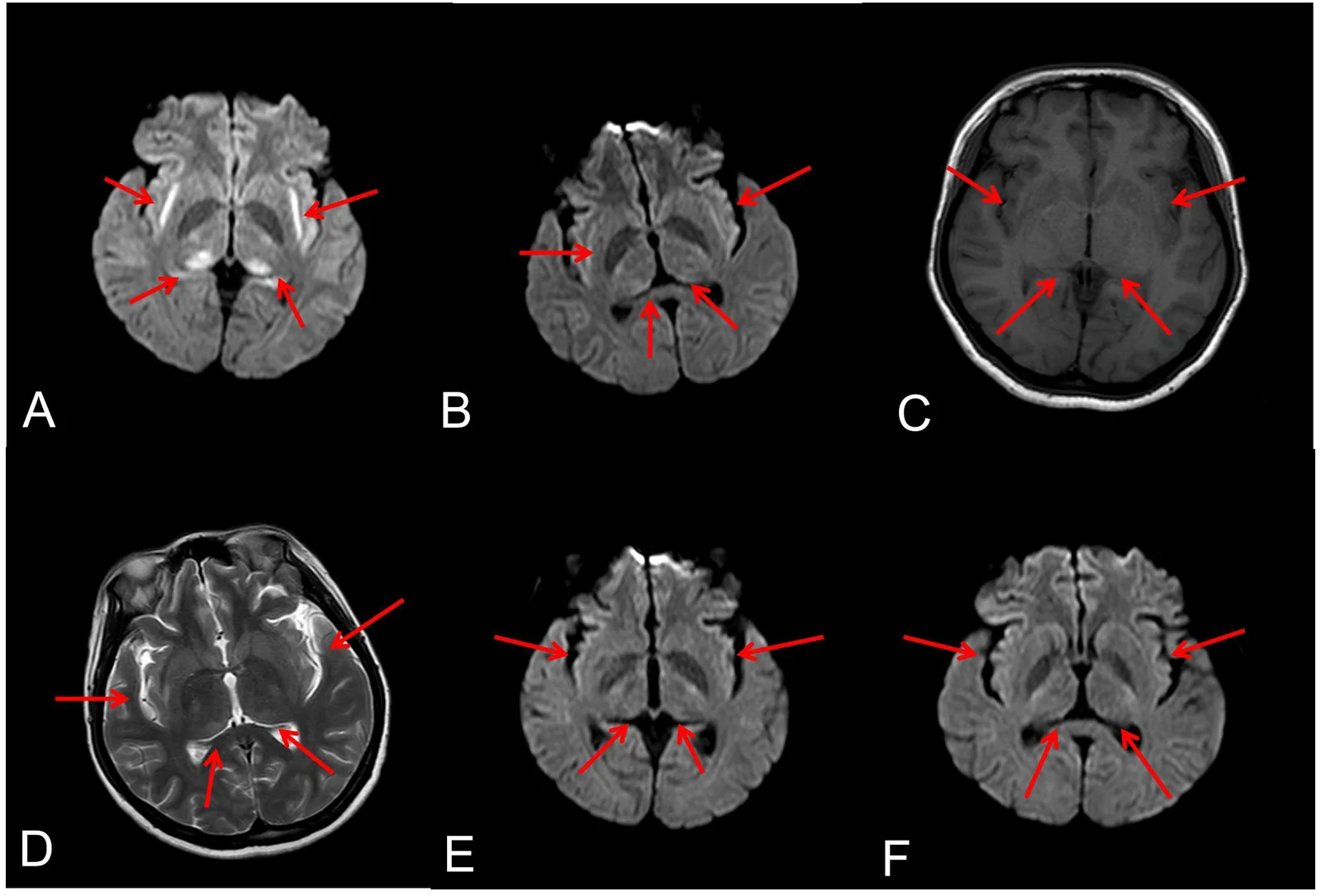
Serial brain magnetic resonance imaging (MRI) during the disease course. **(A–D)** MRI on hospital day 2 (D2) shows bilateral symmetric signal abnormalities in the thalami and claustra: isointense on T1-weighted imaging [T1WI; **(A)**], mildly hyperintense on T2-weighted imaging [T2WI; **(B)**] and T2 fluid-attenuated inversion recovery [T2-FLAIR; **(C)**], and hyperintense on diffusion-weighted imaging [DWI; **(D)**]. **(E)** Follow-up MRI on D24 shows marked improvement of the DWI hyperintensity in the bilateral thalami and claustra. **(F)** MRI at 60 days after discharge shows complete resolution of the DWI hyperintensity. D, hospital day; DWI, diffusion-weighted imaging; MRI, magnetic resonance imaging; T2-FLAIR, T2 fluid-attenuated inversion recovery.

**Figure 3:**
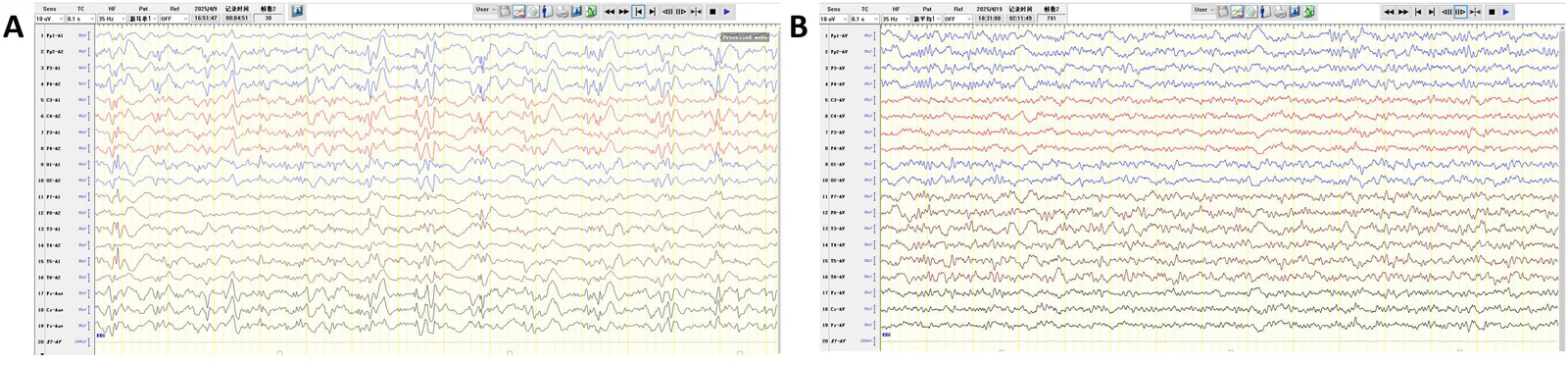
Electroencephalography (EEG) on D4 demonstrated a burst-suppression tendency with quasi-periodic discharges and frequent electrographic seizures arising from the bilateral frontopolar, frontal, and temporal regions, consistent with status epilepticus **(A)**. By D16, the EEG background had improved to predominantly fast and spindle rhythms with preserved stage II–III sleep architecture, and only occasional spike-and-wave discharges remained over the right frontal and temporal regions **(B)**.

**Figure 4:**
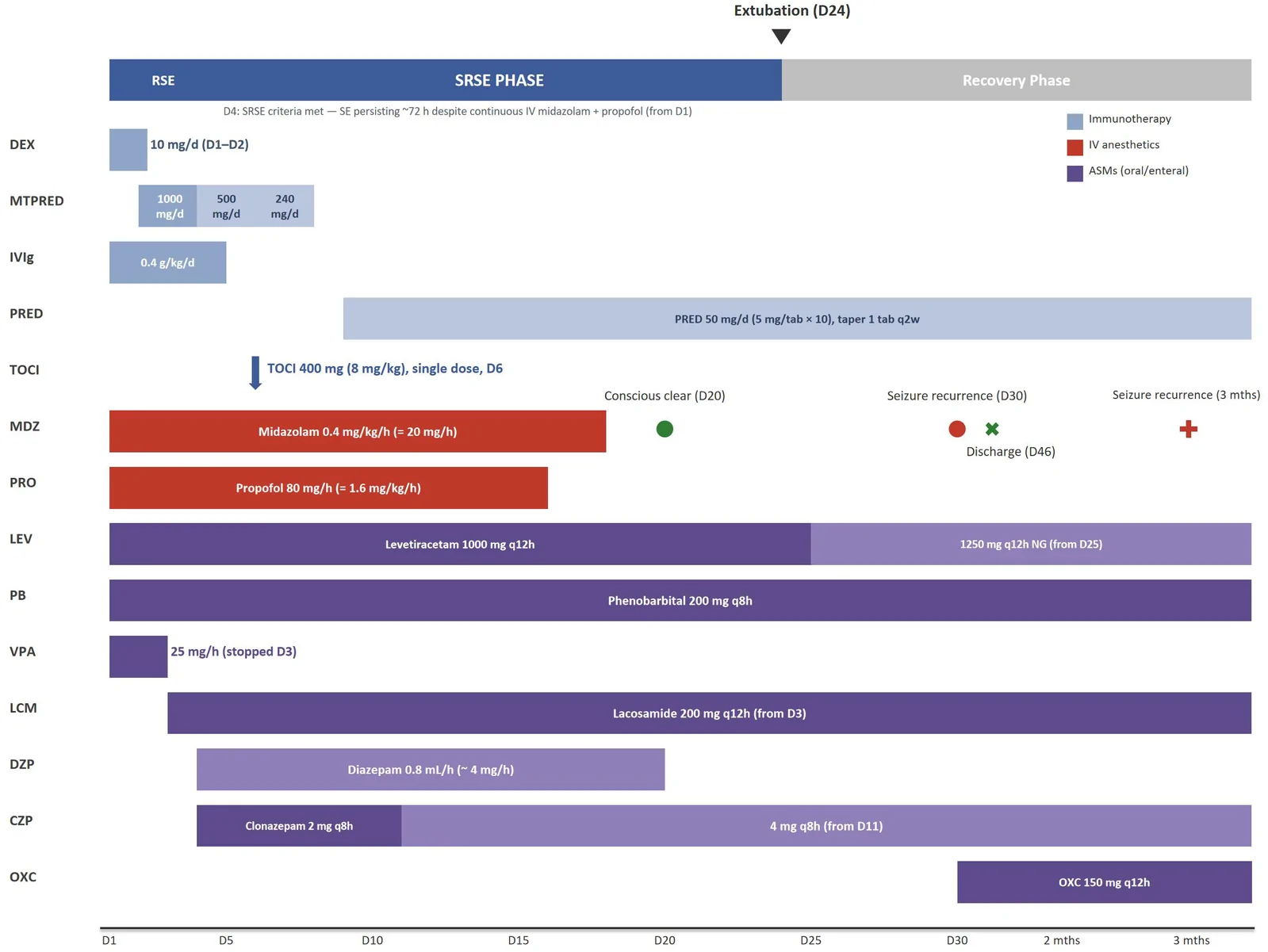
Treatment timeline and clinical events from admission to 3-month follow-up. Light blue, immunotherapy; red, intravenous anesthetics; purple, antiseizure medications (ASMs). The top bar delineates the refractory (RSE), super-refractory (SRSE), and recovery phases of status epilepticus; SRSE criteria were met on day 4 (D4). Immunotherapies included dexamethasone (DEX), methylprednisolone pulse (MTPRED), intravenous immunoglobulin (IVIg), oral prednisone (PRED), and a single dose of tocilizumab (TOCI; 400 mg, 8 mg/kg) on D6 (arrow). Intravenous anesthetics comprised midazolam (MDZ) and propofol (PRO). ASMs included levetiracetam (LEV), phenobarbital (PB), valproate (VPA), lacosamide (LCM), diazepam (DZP), clonazepam (CZP), and oxcarbazepine (OXC). Symbols: ▼, extubation (D24); ●, recovery of consciousness (D20); red ●, seizure recurrence (D30); ✖, discharge (D46); ✚, seizure recurrence at 3 months.

Supportive care included mechanical ventilation, enteral nutrition, fluid resuscitation, antimicrobials (acyclovir; cefoperazone for pulmonary infection), and tracheotomy. On hospital day 1, intravenous dexamethasone (10 mg/day) was administered for 2 consecutive days, and intravenous human immunoglobulin (0.4 g/kg/day) was given for 5 days. Antiseizure therapy was initiated with midazolam (0.4 mg/kg/h continuous intravenous infusion), propofol (80 mg/h continuous intravenous infusion, administered from hospital day 1, tapered from hospital day 11, and discontinued on hospital day 16), sodium valproate (continuous intravenous infusion at 25 mg/h), phenobarbital (200 mg every 8 hours), and levetiracetam (1000 mg every 12 hours) (**Figure 4**). Throughout propofol infusion (hospital days 1–16), the infusion rate (80 mg/h, approximately 1.6 mg/kg/h at a body weight of 50 kg) remained well below the high-risk threshold of 4 mg/kg/h associated with propofol infusion syndrome (PRIS); serum triglycerides, creatine kinase, and lactate were monitored throughout the infusion and remained within normal limits (serial values are not individually reported, as all measurements were unremarkable); no clinical or laboratory signs of PRIS developed. On hospital day 3, sodium valproate was discontinued due to elevated blood ammonia levels, and lacosamide (200 mg every 12 hours) was added. On hospital day 4, electroclinical status epilepticus persisted despite approximately 72 hours of continuous intravenous anesthetic therapy with midazolam and propofol, thereby fulfilling the consensus definition of super-refractory status epilepticus (SRSE)—status epilepticus that persists or recurs 24 hours or more after the onset of anesthetic therapy, including recurrence during anesthetic weaning. After a diazepam bolus produced marked EEG improvement, continuous intravenous diazepam infusion (0.8 mL/h, approximately 4 mg/h with the standard 10 mg/2 mL formulation) was initiated in combination with clonazepam (2 mg every 8 hours). Pulse corticosteroid therapy with intravenous methylprednisolone sodium succinate was initiated at 1000 mg/day, tapered to 500 mg/day on hospital day 4 and further to 240 mg/day on hospital day 6, and completed on hospital day 9, with subsequent transition to oral prednisone acetate tablets 50 mg/day (5 mg per tablet × 10) for 14 days, followed by a tapering regimen of one tablet (5 mg) every 2 weeks. Ketogenic dietary therapy (from hospital day 1) was poorly tolerated and discontinued on hospital day 9 (loose stools, hyperlipidemia, decreased serum calcium and zinc). On hospital day 6, status epilepticus remained refractory. Following comprehensive informed consent, tocilizumab (8 mg/kg, i.e., a total of 400 mg at a body weight of 50 kg) was administered intravenously once. In line with published experience, in which tocilizumab is typically given as a single dose and repetition is reserved for non-responders, a second dose was withheld because electroclinical seizure frequency and CSF IL-6 declined after the first dose amid concurrent immunotherapies. On hospital day 7, the EEG background was unchanged, whereas the frequency of electroclinical seizures had decreased. On hospital day 9, repeat CSF cytokine analysis revealed persistently elevated IL-1β (14.90 pg/mL) and IL-12p70 (4.04 pg/mL), whereas IL-6 had decreased to 14.36 pg/mL (still above the reference upper limit of 5.4 pg/mL); the remaining cytokines were within their reference intervals or below the detection limit. By hospital day 11, electroclinical seizures had decreased to 1–2 episodes per day, the clonazepam dose was increased to 4 mg every 8 hours, and propofol tapering was initiated. By hospital day 13, the patient was free of clinical seizures; the EEG background demonstrated predominant theta activity admixed with abundant fast frequencies and sleep spindles, with occasional sporadic spike-and-slow-wave and sharp-and-slow-wave discharges over the right frontal and temporal regions and preserved stage II–III sleep architecture (**Figure 3B**). Propofol was discontinued on hospital day 16, midazolam on hospital day 18, and diazepam on hospital day 20, when the patient regained full consciousness and was able to follow commands. On hospital day 24, the patient was successfully weaned from mechanical ventilation; follow-up MRI on D24 showed marked improvement of the DWI hyperintensity in these regions (**Figure 2E**). The levetiracetam dose was increased from 1000 mg to 1250 mg every 12 hours on hospital day 25, administered via a nasogastric feeding tube. On hospital day 30, breakthrough seizures recurred—loss of consciousness with left-sided convulsions and leftward conjugate gaze deviation, lasting 1–2 minutes with subsequent recovery—and oxcarbazepine (150 mg every 12 hours) was added. On hospital day 37, the patient was transferred to the general neurology ward, and she was discharged on hospital day 46 without further seizure recurrence. Discharge examination revealed an alert, oriented patient with grade 5/5 strength and absent deep tendon reflexes; the modified Rankin Scale (mRS) score was 0. At the first outpatient follow-up (2 weeks after discharge), gradual tapering of clonazepam and prednisone was continued. At 60-day post-discharge follow-up, the patient experienced one breakthrough seizure during clonazepam dose reduction (left-sided convulsions with leftward gaze deviation, 1–2 minutes, with full recovery). MRI at 60 days after discharge demonstrated complete resolution of the signal abnormalities (**Figure 2F**). Neurocognitive assessment revealed a Mini-Mental State Examination score of 23 and a Montreal Cognitive Assessment score of 21, indicating residual cognitive impairment despite the mRS score of 0; longitudinal follow-up is ongoing.

## Discussion

Human rhinovirus (HRV) demonstrates tropism for airway epithelial cells and induces a broad spectrum of respiratory tract infections (1); central nervous system involvement remains exceedingly rare( (2–4). In the present case, HRV was detected only in an upper respiratory tract specimen (nasopharyngeal swab) during the acute illness, whereas the cerebrospinal fluid (CSF) tested negative by targeted next-generation sequencing (tNGS; a panel covering 429 organisms that specifically includes rhinoviruses A–C). These negative results cannot exclude trace virus below the detection limit, and asymptomatic carriage with respiratory shedding for several weeks is well documented (4, 5); detection in a single respiratory specimen therefore lacks specificity for central nervous system (CNS) infection. The available evidence thus supports only a temporal association between HRV detection and the development of FIRES, rather than a direct causal relationship, and a cytokine-mediated neuroinflammatory mechanism—rather than direct viral neuroinvasion—remains a plausible but unproven hypothesis (6–8). Critically, because HRV circulates widely and asymptomatic respiratory shedding is common, the possibility that HRV was detected in this patient by chance—representing asymptomatic carriage coinciding with an unrelated febrile or non-infectious process—cannot be excluded; this possibility is explicitly acknowledged as a fundamental limitation of the present report.

According to the 2018 international consensus definitions (9), febrile infection-related epilepsy syndrome (FIRES) is a subcategory of new-onset refractory status epilepticus (NORSE) in which refractory status epilepticus (RSE) arises without an identifiable acute structural, toxic, or metabolic cause and is preceded by a febrile infection beginning between 24 hours and 2 weeks before seizure onset (10). This definition rests on temporal association rather than proven causality; consistent with this, no etiology is ultimately established in more than half of NORSE cases despite extensive testing (9, 10). Our patient fulfills these criteria: seizures began 3 days after fever onset, progressed through RSE to super-refractory status epilepticus (SRSE) (11), and no structural, toxic, metabolic, or genetic etiology was identified. As HRV was detected only in respiratory specimens without evidence of CNS invasion, we consider it a possible para-infectious peripheral trigger of an immune-mediated process. Because the seizures began on illness day 4, while the febrile illness was still ongoing, the temporal relationship is more accurately described as a syndrome temporally associated with an HRV-positive febrile respiratory illness (i.e., para-infectious) rather than post-infectious (12, 13), immune-mediated process and describe this patient as having FIRES with claustral involvement (i.e., the “claustrum sign”) (14–16).

Published FIRES/NORSE cases with claustral involvement share four recurrent features (3, 15, 17): (1) acute onset of neuropsychiatric manifestations during or shortly after a systemic febrile infection; (2) absent or mild CSF pleocytosis with elevated IL-6 levels in CSF and/or serum and negative autoimmune encephalitis antibodies; (3) reasonable exclusion of alternative etiologies; and (4) bilateral claustral signal abnormalities on MRI, sometimes with hippocampal involvement. These features are a descriptive synthesis of previous reports, not validated diagnostic criteria. The secondary seizures are acute symptomatic seizures driven by inflammatory-cascade activation and pro-inflammatory cytokine release, mechanisms common to CNS infections (18, 19). Our findings are compatible with the concept that FIRES pathogenesis involves a sequential process: an initial peripheral inflammatory trigger followed, after a latent interval, by a central neuroinflammatory cascade that drives refractory seizure activity (20, 21). Consistent with this model, the admission CSF IL-6 level (121.93 pg/mL) exceeded the paired serum level (97.1 pg/mL), suggesting compartmentalized intrathecal inflammation and rendering direct viral invasion of the CNS less likely—although not excluding it. The claustrum may additionally serve as an anatomical hub mediating the propagation and synchronization of epileptiform discharges across cortical regions (22); inflammation-induced claustral dysfunction could therefore facilitate rapid seizure generalization (14). Conversely, seizure activity per se can precipitate transient, reversible MRI signal alterations, and the complete resolution observed in our patient leaves open whether the claustral lesions were primary or secondary to status epilepticus (23). Finally, the symmetrical bilateral claustral and thalamic DWI hyperintensities warrant explicit consideration of acute necrotizing encephalopathy (ANE, including RANBP2-related ANE), artery-of-Percheron infarction, Wernicke encephalopathy, and peri-ictal changes. The epilepsy gene panel covered RANBP2 and detected no clinically relevant variants, and follow-up imaging showed no necrotic evolution, arguing against ANE. Artery-of-Percheron infarction was unlikely given the strict symmetry, the additional claustral involvement beyond a single perforator territory, and the complete resolution; magnetic resonance angiography (MRA) was not performed (acknowledged as a limitation). Wernicke encephalopathy was considered unlikely given the absence of nutritional-deficiency risk factors and the resolution without thiamine. Apparent diffusion coefficient maps were not acquired, and susceptibility-weighted imaging and post-contrast sequences were not performed; these gaps are likewise acknowledged as limitations.

In previously reported cases of FIRES/NORSE with claustral involvement, a prodrome of fever and altered mental status is followed after several days by seizures, and claustral lesions typically become radiologically apparent one to two weeks after onset (16); these observations derive from a small number of cases and are presented as a narrative rather than a systematic review. Seizures evolve rapidly to generalized non-convulsive or subtle status epilepticus, usually requiring neuro-intensive care and intravenous anesthetics (24). Claustral signal changes resolved after control of status epilepticus in most reported cases, although varying degrees of cortical and/or hippocampal atrophy may follow, and all reported patients tested negative for known autoimmune encephalitis antibodies (25). First-line immunomodulatory therapy (corticosteroids, intravenous immunoglobulin) is recommended within 72 hours of status onset and may improve neurological outcomes. Ketogenic dietary therapy is recommended as an early adjunct because of its combined antiseizure and anti-inflammatory effects (26); in our patient it was discontinued for intolerance and alternative formulations were not pursued (acknowledged as a limitation). Tocilizumab and anakinra represent the principal second-line options, with a growing trend toward earlier implementation (27). In our patient, clinical and electrographic improvement followed multimodal treatment that included tocilizumab (administered on hospital day 6); however, because corticosteroids, immunoglobulin, multiple antiseizure medications, and continuous anesthetic agents were administered concurrently or sequentially (**Figure 4**), the contribution of tocilizumab cannot be isolated, and the temporal association should not be interpreted as evidence of efficacy. Nor can the CSF IL-6 decline be attributed to tocilizumab, given concomitant immunomodulation and the natural evolution of FIRES; tocilizumab characteristically increases circulating IL-6 by blocking receptor-mediated clearance, so the observed serum decline (from 97.1 to 56.8 pg/mL) may reflect assay characteristics, sampling timing, or concurrent high-dose corticosteroids; and the essentially unchanged IL-1β (from 14.38 to 14.90 pg/mL) and IL-12p70 (from 4.16 to 4.04 pg/mL) levels indicate that innate immune hyperactivation had not fully resolved. In addition, the serum and CSF sampling time points were not matched (CSF: hospital days 2 and 9; serum: days 1 and 16); the two compartments should therefore be interpreted separately, with all concomitant treatments taken into account at each time point. Although the admission CSF IL-6 level (121.93 pg/mL) was clearly above the laboratory reference interval (upper limit 5.4 pg/mL), it lies at the lower end of the range reported in NORSE cohorts and in severe viral encephalitis and should not be regarded as exceptional (28); given the limited number of measurements, no meaningful quantitative correlation between serum IL-6 levels and seizure frequency can be established. Finally, the acute-phase EEG findings—including the burst-suppression tendency—were obtained under continuous midazolam and propofol infusion together with multiple antiseizure medications and should be interpreted accordingly; continuous EEG monitoring aids antiseizure drug selection, titration, and discontinuation decisions, and immediate cessation of ongoing seizure activity remains the primary therapeutic objective (10, 29).

## Conclusions

In patients presenting with neuropsychiatric manifestations, epileptic seizures, and altered consciousness following an HRV-positive febrile respiratory illness, clinicians should maintain a high index of suspicion for FIRES with claustral involvement temporally associated with the infection. Cranial MRI combined with CSF analysis and comprehensive neurological assessment provides critical evidence for early identification. However, the absence of detectable HRV in the CSF argues against—without excluding—direct viral neuroinvasion, and a cytokine-mediated neuroinflammatory mechanism remains a hypothesis consistent with the observed intrathecal IL-6 elevation rather than a proven mechanism. Early antiseizure treatment combined with intensive multimodal immunotherapy, optimized under continuous EEG monitoring, may interrupt the vicious cycle between epileptic activity and neuroinflammation and was followed by clinical and electrographic improvement in this patient; however, because multiple therapies were administered concurrently or sequentially, the contribution of any specific treatment, including tocilizumab, cannot be determined from a single case, and no conclusions can be drawn regarding the efficacy of tocilizumab or the clinical utility of cytokine monitoring. Serum and CSF cytokine profiling may provide mechanistic insight when obtained promptly alongside cranial MRI, but its value for guiding therapy remains investigational; structured long-term follow-up remains necessary given the residual cognitive impairment.

## Data Availability

The original contributions presented in the study are included in the article/**Supplementary Material**. Further inquiries can be directed to the corresponding author.
