# Supplementary material for: Febrile infection-related epilepsy syndrome with claustrum sign following a human rhinovirus-positive respiratory illness in an adult: a case report
Source: Front Immunol. 2026 Sep 17;17:1935305. doi: 10.3389/fimmu.2026.1935305 (PMC13627439; doi:10.3389/fimmu.2026.1935305)
Supplement: Supplementary file 1 [file Table1.docx]

**Supplementary Material**

**Supplementary Table S1. Microbiological and virological investigations.**

| **Specimen** | **Collection (hospital day)** | **Assay (method, manufacturer)** | **Pathogens covered** | **Result** |
| --- | --- | --- | --- | --- |
| Nasopharyngeal swab | Day 1 (illness day 9) | Multiplex real-time fluorescent RT-PCR; Respiratory Six-Virus Nucleic Acid Detection Kit (Shanghai BioGerm Medical Technology Co., Ltd., Shanghai, China) | HRV, human parainfluenza virus types 1 and 3, human adenovirus, human metapneumovirus, respiratory syncytial virus | HRV detected; other five panel viruses negative |
| Sputum | Day 1 | Same multiplex real-time fluorescent RT-PCR panel (as above) | As above | Negative for all six panel viruses (including HRV) |
| Cerebrospinal fluid | Day 2 (April 7, 2025) | Targeted next-generation sequencing (tNGS): ultra-multiplex PCR enrichment plus high-throughput sequencing; Common Pathogens Targeted Identification Panel (400+ targets and drug-resistance genes); Wuhan Kindstar Zhenyuan Medical Laboratory, Wuhan, China | 429 organisms: bacteria (114 Gram-positive and 83 Gram-negative species), fungi (71 species), DNA viruses, RNA viruses (66 species, including HSV-1/2, VZV, EBV, CMV, HHV-6–8, enteroviruses A–D, echoviruses, rhinoviruses A–C), parasites; plus antibiotic-resistance and virulence genes; lower limit of detection 100 copies/mL | No pathogenic microorganisms detected; no grey-zone or background signals; no resistance or virulence genes detected |
| Blood culture | Admission | Routine aerobic and anaerobic culture | Bacteria and fungi | Negative |
| Serum | Admission | TORCH panel | Toxoplasma gondii, rubella virus, CMV, HSV-1/2 | Unremarkable |
| Skin test | Admission | Purified protein derivative (PPD) test | Mycobacterium tuberculosis | Negative |

*CMV, cytomegalovirus; CSF, cerebrospinal fluid; Ct, cycle threshold; EBV, Epstein-Barr virus; HSV, herpes simplex virus; RT-PCR, reverse-transcription polymerase chain reaction; TORCH, Toxoplasma–rubella–CMV–HSV panel; VZV, varicella-zoster virus. Real-time fluorescent RT-PCR results were interpreted as positive when Ct ≤ 35 with a typical sigmoidal amplification curve, equivocal when Ct 36–38 (retested), and negative when Ct > 38 or undetected.*

**Supplementary Table S2. Autoantibodies investigated in paired serum and CSF specimens.**

| **Category** | **Antibody** | **Serum** | **CSF** |
| --- | --- | --- | --- |
| Autoimmune encephalitis (16-item panel) | Anti-NMDAR antibody (anti-glutamate receptor, NMDA type) | Negative | Negative |
| Autoimmune encephalitis (16-item panel) | Anti-AMPAR1 antibody (anti-glutamate receptor, AMPA 1 type) | Negative | Negative |
| Autoimmune encephalitis (16-item panel) | Anti-AMPAR2 antibody (anti-glutamate receptor, AMPA 2 type) | Negative | Negative |
| Autoimmune encephalitis (16-item panel) | Anti-GABABR antibody | Negative | Negative |
| Autoimmune encephalitis (16-item panel) | Anti-LGI1 antibody | Negative | Negative |
| Autoimmune encephalitis (16-item panel) | Anti-CASPR2 antibody | Negative | Negative |
| Autoimmune encephalitis (16-item panel) | Anti-IgLON5 antibody | Negative | Negative |
| Autoimmune encephalitis (16-item panel) | Anti-DPPX antibody (anti-dipeptidyl-peptidase-like protein 6) | Negative | Negative |
| Autoimmune encephalitis (16-item panel) | Anti-D2R antibody (anti-dopamine D2 receptor) | Negative | Negative |
| Autoimmune encephalitis (16-item panel) | Anti-GAD65 antibody (anti-glutamic acid decarboxylase 65) | Negative | Negative |
| Autoimmune encephalitis (16-item panel) | Anti-GlyRα1 antibody (anti-glycine receptor α1 subunit) | Negative | Negative |
| Autoimmune encephalitis (16-item panel) | Anti-mGluR1 antibody (anti-metabotropic glutamate receptor 1) | Negative | Negative |
| Autoimmune encephalitis (16-item panel) | Anti-mGluR5 antibody (anti-metabotropic glutamate receptor 5) | Negative | Negative |
| Autoimmune encephalitis (16-item panel) | Anti-GABAARα1 antibody (anti-γ-aminobutyric acid A receptor, α1 subunit) | Negative | Negative |
| Autoimmune encephalitis (16-item panel) | Anti-GABAARβ3 antibody (anti-γ-aminobutyric acid A receptor, β3 subunit) | Negative | Negative |
| Autoimmune encephalitis (16-item panel) | Anti-Neurexin-3α antibody | Negative | Negative |
| Demyelination-associated / astrocytopathy-related (3-item panel) | Anti-AQP4 antibody (anti-aquaporin-4) | Negative | Negative |
| Demyelination-associated / astrocytopathy-related (3-item panel) | Anti-MOG antibody (anti-myelin oligodendrocyte glycoprotein) | Negative | Negative |
| Demyelination-associated / astrocytopathy-related (3-item panel) | Anti-GFAP antibody (anti-glial fibrillary acidic protein) | Negative | Negative |

*CSF, cerebrospinal fluid. All antibodies were tested by cell-based assay (CBA) with fluorescence microscopy on paired serum and CSF specimens collected on hospital day 2 (April 7, 2025), at an external reference laboratory (Beijing Hightrust Diagnostics Co., Ltd., Beijing, China); results were reported on April 8, 2025. These panels comprise all antibodies sent for testing during the hospitalization.*

**Supplementary Table S3. Systematic exclusion of alternative etiologies.**

| **Category** | **Investigations** | **Result** |
| --- | --- | --- |
| Structural | Cranial CT (admission) | Unremarkable |
| Structural | Brain MRI D2 (T1WI, T2WI, T2-FLAIR, DWI) | Bilateral symmetric thalamic and claustral signal abnormalities |
| Structural | Follow-up MRI D24; MRI 60 days after discharge | Marked improvement (D24); complete resolution (60 days) |
| Structural (limitations) | MRA, SWI, post-contrast sequences, ADC maps | Not performed (acknowledged as a limitation) |
| Toxic | Clinical history: alcohol, recreational substances, medications, and occupational/environmental exposures | No history of alcohol misuse, substance use, or toxic exposure was elicited; no toxic cause identified |
| Metabolic | Complete blood count; thyroid function; inherited metabolic screening | Unremarkable |
| Infectious | See Supplementary Table S1 (nasopharyngeal swab and sputum multiplex RT-PCR, CSF targeted next-generation sequencing panel, blood cultures, TORCH panel, PPD test) | No pathogenic microorganisms detected in CSF; HRV detected in respiratory specimen only |
| Autoimmune/inflammatory | Autoantibody panels (19 antibodies; Supplementary Table S2); CSF routine, biochemistry, cytology, immunoglobulin G | All 19 antibodies negative in paired serum and CSF; opening pressure 230 mmH₂O; mild pleocytosis (12×10⁶/L); CSF immunoglobulin G 22.10 mg/g |
| Paraneoplastic | 16-item autoimmune encephalitis panel including multiple paraneoplastic-associated antibodies (e.g., anti-AMPAR1/2, anti-GABABR, anti-CASPR2, anti-IgLON5, anti-DPPX, anti-GAD65, anti-mGluR1, anti-mGluR5; Supplementary Table S2); serum tumor markers (CEA, AFP, CA19-9, CA72-4, CA125, HE4, CA15-3); chest CT | All antibodies negative; all seven serum tumor markers negative; chest CT showed bilateral pulmonary inflammatory infiltrates without evidence of tumor |
| Genetic | Epilepsy gene panel (target-region capture, DNBSEQ-T7 sequencing, proband only) | No clinically relevant single-nucleotide, copy-number, or mitochondrial variants (RANBP2 covered) |
| Vascular | Clinical-radiological assessment for artery-of-Percheron infarction | Unlikely (strict symmetry, claustral involvement beyond a single perforator territory, complete resolution); MRA not performed |
| Nutritional | Clinical assessment for Wernicke encephalopathy | Unlikely (no risk factors; resolution without thiamine supplementation) |

*ADC, apparent diffusion coefficient; CBC, complete blood count; CSF, cerebrospinal fluid; DWI, diffusion-weighted imaging; MRA, magnetic resonance angiography; SWI, susceptibility-weighted imaging; TORCH, Toxoplasma–rubella–CMV–HSV panel.*
